# Promoting Physical Activity in Group Home Settings: Staff Perspectives through a SWOT Analysis

**DOI:** 10.3390/ijerph17165805

**Published:** 2020-08-11

**Authors:** Bik C. Chow, Peggy Hiu Nam Choi, Wendy Yajun Huang, Chien-yu Pan

**Affiliations:** 1 Department of Sport and Physical Education, Hong Kong Baptist University, Hong Kong; wendyhuang@hkbu.edu.hk; 2 Department of Sports and Recreation, Technological and Higher Education Institute of Hong Kong, Hong Kong; pchoi@vtc.edu.hk; 3 Department of Physical Education, National Kaohsiung Normal University, Kaohsiung 80201, Taiwan; chpan@nknu.edu.tw

**Keywords:** focus group, group home, health promotion, intellectual disabilities, physical activity

## Abstract

Purpose: The aim of the study was to investigate perceptions of staff about the promotion of physical activity (PA) in selected group residences of Hong Kong (HK), some of which had experienced a multi-component PA program. Method: Focus group interviews with nineteen staff members from four group homes (two of which received the program) were conducted. Findings: A SWOT analysis provided important insights into residential staff views about key influences on the quality of PA programs for residents with intellectual disabilities (ID). Positive (strengths and opportunities) and negative (weaknesses and threats) influences were identified. They were associated with characteristics of residents, staff, and group residence. Increasing age and low motivation are impediments to PA engagement of adults with ID. Staff competence and prior unsuccessful experience in promoting PA are also implicated. Conclusion: The PA program quality is mediated by the quality of staff interpersonal interactions with their clients and their commitment in encouraging such adults with ID to join and persistent in PA as well as staff seeking external resources and support as well as using initiative to adapt PA promotion activities in their specific group residential context.

## 1. Introduction

Hong Kong (HK) is a highly urbanized modern city, easily recognized for the density of high-rise buildings that occupy only 25% of the land mass [1]. The open areas for hiking are accessed by car or public transport. The HK population is essentially homogeneous: 92% ethnic Chinese [2]. The prevalence of people with intellectual disabilities (ID) in HK is between 1.0% and 1.4% (71,000–101,000) [3]. Before the age of 18, a person displaying significant limitations in two or more areas of adaptive behavior on learning of conceptual, social, and daily living skills is clinically diagnosed with ID [4]. Unfortunately, when compared with the general population, adults with ID are often at greater risk of mortality and morbidities partly due to their health status. However, with medical advancements, the average lifespan of adults with ID has significantly increased in recent decades [5].

Evidence shows that adults with ID have generally increased vulnerability to poor health and physical illness, such as obesity and chronic diseases [6]. To combat the adverse effects of chronic diseases, health promotion initiatives through physical activity (PA) have been advocated worldwide [7]. Despite well-established research evidence that documents the benefits of leading a physically active lifestyle, adults with ID have extremely low PA levels; for example, only 9% of such individuals met PA guidelines set by the World Health Organization (specifically, 150 min per week of moderate-to-vigorous PA), compared with 30% to 47% of the population without disabilities [8]. Physical inactivity is detrimental to health because sedentariness is an independent risk associated with adverse health [9]. Hence, health researchers have urged more PA programs targeted at this special population. However, very few PA or exercise intervention programs have been offered to adults with ID in Asia countries. A recent systematic review [10], searching databases until July 2017, found nine PA intervention studies. These were of randomized controlled trials in people with ID. None of those studies was conducted in Asian countries. When including non-randomized controlled trials in exercise intervention studies on the physical fitness level of adults with ID (searched database up to January 2018) Bouzas et al. [11] identified 44 studies, only three of which were conducted in east Asia (South Korea–2 and Taiwan–1); all others (93%) were conducted in western countries.

People with ID are particularly vulnerable to their residential circumstances because they often rely on support for daily routines [12]. Studies have found specific residential settings associated with variable health outcomes [13] and quality of life [14]. Temple and Walkley [15] found physical inactivity as being related to more restrictive settings and lower abilities (conceptual, social, and daily living). Supported services in residential environments can be a key determinant of PA participation for adults with ID [16]. In HK there are 132 group homes offering 24 h care predominantly for people with ID aged 15 years and above (*n* = 7444) of which 31 are for mild and moderate ID persons (*n* = 1745, mean = 53 residents per home) [17]. Fifty-two percent of such residences are paired with sheltered workshops, and their residents (*n* = 939) are eligible for full-time employment. These group homes are administered by non-government agencies with governmental subsidies. In addition, 4947 people with moderate and severe ID are on waiting list to be admitted into a group home [18]. A literature review suggests that there is no documented study about how this dimension of the HK population responds to PA promotion and what strategies might be effective in residential settings in this city. In particular, group homes for people with ID in the western context are referred to a licensed community residential setting for four to eight persons, supervised by paid staff [19]. However, in HK, group homes for adults with ID are relatively larger (resident mean exceeding 50). By virtue of HK’s building density, its contextual factors for group homes vastly differ from those in the West.

Dixon-Ibarra [20,21] reported barriers (resident motivation, limited staff, negative support, and resident age) and facilitators (role modelling/encouraging, enjoyment). A recent qualitative study [22] focused on motivational factors of PA participation perceived by health care workers and family members; its findings suggested that engagement with contextual support is a prerequisite for higher motivation. Other research findings have indicated that the characteristics of the residents (e.g., physical and mental health, age, preference/motivation) could have impacted on the capability, motivation, and opportunity of the professional staff in rendering PA support for people with ID [16]. Findings from a qualitative study focusing on residents’ feedback after receiving health promotion programs showed that adults with ID had increased health awareness of healthy lifestyle [23]. From our review, no qualitative study has been conducted on the promotion of PA in the group homes of HK. In diversifying the research knowledge base, **t**he findings could have important implications for health promotion researchers, policy makers, and practitioners, to enable them to plan effective strategies when designing PA intervention programs for people with ID living in group residences [21,23]. This qualitative study focused on PA among HK people with ID in residential care. It was intended to investigate the perceptions of staff about the promotion of PA in group homes, some of which had been involved in a multi-component PA program.

## 2. Method 

### 2.1. Sample Selection and Description

Four group homes (GA), two (GHA and GHB) having received a PA program intervention and two without (GHC and GHD) were purposely selected through reputations of best practice in the field of residential care for people with ID. No site had previously received PA promotion support in the form of program intervention from external agencies such as Special Olympics HK. None of the homes was located near the open recreation areas of HK. Managers of each group home were asked to invite three to six staff members for an interview.

Informed consent and permission for interviewing staff and audiotaping were obtained. Participants were informed of the study’s purpose and were assured of anonymity and confidentiality. Interviews took place in the multi-purpose rooms of each group home. All interviews were conducted by the first two authors (both female); the lead author has research and interview experience in qualitative studies, and the second author has pedagogical expertise in adapted PA. She has extensive teaching and supervisory experience in planning and presenting physical activities for people with ID.

At GHA and GHB, the authors had previously conducted a PA promotion program with two to four months post-intervention review. The intervention included structured exercise training, Social Cognitive Theory-based educational talks for residents, and staff training. The experimental study protocol was reported [24]. GHA and GHB are governed by the same non-government organization while the last two group homes have a different managing organization. Moreover, GHA and GHB have been in operation longer than GHC and GHD. They had relatively fewer residents and had less PA space and total area than the other two group homes (see Table 1). Therefore, the first two group homes and last two group homes are respectively classified as (a) older-smaller homes (OH) and (b) newer-larger homes (NH). Table 1 shows basic demographics in terms of characteristics of residents (population and age) and the sites (location, PA venue, and size).

### 2.2. Data Collection

Focus group interviews were conducted among staff members of group homes; the sessions with three to five interviewees per group followed a semi-structured guide. Questions focused on four broad areas: (a) residents’ health status and their activity/inactivity patterns, (b) concerns and influencing factors related to the residents’ PA participation, (c) experiences in offering physical activities to the residents, and (d) post-intervention program feedback (applicable only to GHA and GHB). 

The interviews (*n* = 4) with a total of 19 staff (female = 9, male = 10) at 4 group homes were conducted between May and November 2016. The total sample comprised managers (*n* = 4), social workers (*n* = 3), caretakers (*n* = 3), program workers (*n* = 4), dormitory managers (*n* = 2), nurses (*n* = 2), and one physiotherapist. Their average work experience at the relevant site was 7.3 years (range: 1 to 20 years, mean for OH = 10.6 years, mean for NH = 3.7 years). The mean interview duration was 43 min (ranged from 30 min to 50 min). A project assistant (a sport studies university graduate) transcribed and translated the interviews into English, and checked for sense by lead authors. 

### 2.3. Data Analysis

Transcript processing involved inductive content analysis [25]. Line-by-line analysis of each transcript was carried out. Preliminary independent analysis by the lead authors determined the general and specific issues voiced by the participants. The researchers (who had been involved in the PA program design, implementation, and evaluation) saw the potential of a “halo effect” [26]. Thus, to remain aware of their own subjectivities [27], they engaged a “critical friend” (CF) in the data analysis and writing process. Coined in 1975, the term “critical friend” [28] has become synonymous with the otherwise uninvolved person who helps a qualitative research team become aware of potential problems in the research process. In this case of the researchers having also been PA intervention program designers, implementers and/or evaluators, we used a CF to identify potential issues, specifically of bias, and thus substantiate trustworthiness of the findings. CF, a retired professor with experience in teaching and conducting qualitative research had been part of neither the PA promotion nor the evaluation team. In order to reduce issues of bias, CF questioned the authors about the developing analysis and critiqued the trustworthiness of their unfolding findings.

The researchers collapsed the initial open coding into four mega themes. This essentially constituted a Strengths, Weaknesses, Opportunities, and Threats (SWOT) analysis. SWOT analysis has its origin in strategic planning and first appeared in the literature in early 1960s [29]. It has been adopted in qualitative analysis in health-related studies in recent years, including policies and programs for diabetes sufferers [30], patient safety curriculum [31], nurse views on curriculum development [32], and strategies for future of healthcare [33]. In the present analysis, a SWOT analysis was conceptualized to evaluate the factors influencing the PA promotion. Studies by Giusti et al. [30] and Gretzky [34] showed that “Strengths” and “Weaknesses” are internal factors (within the scope and control of the organization), respectively, for positive and negative influences. “Opportunities” are external factors (outside the direct control of the organization) for positive growth, and “Threats” are external factors negatively affecting performance.

The researchers then, in accordance with the principles of constant comparison [35], back-checked for similarities and differences. This enabled researchers either to verify an instance that was unique in a case or a dimension of the ID sample (home context) or to ascertain the universality of significance across the four sites of interest.

## 3. Findings

From the SWOT analysis, it is evident that there were positive (strengths and opportunities) and negative (weaknesses and threats) influences on the quality of PA promotion in the sample group homes. Thematic findings based on those etic themes (*Strengths*, *Weaknesses*, *Opportunities*, and *Threats*) are reported below.

### 3.1. Strengths

#### 3.1.1. Staff Commitment

Staff commitment to engaging their residents in PA was seen in two group homes where they had been kicking a soccer ball along corridors and an outdoor court and shooting basketballs with their residents. In addition, group home staff reported persistence in prompting an active lifestyle, among their residents (GHC). When engaging residents in outdoor basketball, staff members were required to accompany and play with their residents to ensure for their safety. Staff reported the perceived benefits of playing sport to developed self-discipline; for example, *“*when taking turns in playing basketball” (GHC). Another staff member mentioned the need for creativity in catering for different abilities and had over a period of time reduced monotony repetition in teaching a resident with reduced mobility to effectively kick in stages: from a “seated position … then … in supported standing position” (GHC).

#### 3.1.2. Motivating Strategies

Staff reported using motivating strategies such as music and the playful nature of some physical activities to engage their clients in PA. As one staffer specifically commented, people with ID were seen to enjoy music. Most residents also perceived exercise as being game-like. Therefore, staff acknowledged that, in order to increase the resident’s interest, game-like rhythmic activities using different toys could be incorporated into the exercise program. Participants’ motivation at GHD was commonly stimulated verbally, encouraging their residents to train hard for their sports day with the prospect medal awards. 

#### 3.1.3. Social Influence

Social influence and positive reinforcement were also seen as means to help residents become more active. In regard to the social interaction between staff and residents, the executive officer of the GHD stated:

We hope to get inter-personal influence to enrich the exercise atmosphere. Therefore, in the initial phase of [a] PA program, we need our staff to lead, guide, and motivate the residents to do the activities. To give exaggerated praises, not saving [with-holding] verbal cheers, we hope our residents will be focused into doing the exercise.

(GHD)

#### 3.1.4. Daily Routine

Staff also recognized the potential contribution of daily routines and tasks to PA engagement. As embedded in the residents’ daily life, adults with ID had to walk up and down stairs and do some simple household chores. As commented by a staffer (GHB), “In general, they can do [physical activities]; our principle is to allow them to do as much as possible, like washing dishes, folding clothes”.

The residential context has potential in either facilitating or impeding the PA levels of the residents. Having sufficient space to incorporate an outdoor area was a group home strength. At NHs, there are outdoor physical activities such as basketball (GHC) and jogging training for GHD’s younger-age residents within its outdoor sport facility. In addition, residential policy to offer a range of physical activities had positive influences on PA promotion. The offered activities included daily morning exercise, regular walking trips to supermarket or community, occasional weekend hiking trips, short-term group programs (lion dance, taekwondo, Thai boxing, video games of bodily movements, ball games training). (See Table 2 for listing of activities.) The following interview statements illustrate policy-related facilitators of PA and their impact on residents’ PA.

*For regular physical activities on Saturday or Sunday, seated Tai-chi is now offered to replace for fitness training because … [it] is more suitable for lower ability ones and is easier to manage discipline. [Required] to join, residents like it because they engage actively. Sometimes staff are prompted by the residents to make preparation for the exercise class.*

(GHA)

*Daily after-breakfast video-exercise is offered. [It’s] 20 min in duration and is mandatary because dormitory rooms are locked. The majority … move [with] the video. This policy was a contrast to their past failed experience to organize a walking program between bath time and dinner time. But residents hid themselves in their dormitory rooms.*

(GHB)

*We have organized an exercise video-session on Wednesday night for all residents for a month now, and [it] will be run as a regular event. We hope this session (involving group dancing) … [will be a] social influence so that they will move more.*

(GHD)

At GHD, those residents who were usually PA refusers were encouraged “to walk to supermarket because walking is still a type of exercise. It’s once a week and will give priority to those who stay at dormitory during weekends” (GHD).

The two and a half hours between dinner and bedtime was another opportunity to offer physical activities. This practice was specific to GHD where residents were younger (20–30 years old); activities included Taekwondo (particularly well received by residents who reportedly liked Kung-fu drama) and a group fitness training program. GHD purposely selected a public swimming pool because it required 40 min travel time and gave an opportunity for their residents to get into a different community on the weekend. There was seasonal variation in planning weekend activities: swimming was offered in summer and hiking in autumn.

Establishing institutional policy about daily routines was seen as essential in promoting residents’ habitual PA. In order to increase PA, GHC required their residents to use stairs to access the dinning floor. GHA’s daily morning exercise was mandatary, held at a time when dormitory bedrooms were locked. In addition, institutional review provided opportunity for reflection on the effectiveness of current PA programs and for future planning (GHD): Staff recommended a weekly “exercise for all” program prompted by a movement video.

#### 3.1.5. Staff Development

Staff from GHA and GHB participated in a multi-component PA program led by the authors. The program was viewed as having fun with participants (residents and staff) enjoying it. When asked about the effectiveness of the program, a staffer commented, “I feel that it can build up [residents] habit of being physically active”. Another reflected, “I can say they felt novel and freshness towards physical exercise. Also having music in the lessons made them more immersed into the exercise”. The professional development program had broadened staff knowledge about instructing a group exercise class. The use of a warm-up routine, music, and toys was considered instrumental in prompting movement among the participants. Staff also perceived three information sessions as being useful because they observed that those residents of higher cognitive ability had openly enjoying them and appropriately responded to instructions. Moreover, issuing stickers as a reward for correct answers encouraged the residents. In terms of one-hour staff training, personnel indicated that it was useful professional development especially in providing ideas for motivating games and using equipment in muscle strengthening exercise.

### 3.2. Weaknesses

Unfortunately, the staff perceived many drawbacks to their residents being physically active.

#### 3.2.1. Resident Perceptions

The most frequently cited reasons for resident non-participation in PA were their apparent disinterest in getting moving and low self-efficacy in physical exertion. When mandatory PA sessions were imposed, disinterested residents would refuse. They preferred sedentary activities; the compulsory PA was perceived as “hard to do” and “getting tired”. Staff members from GHB and GHD specifically noted that their residents only wanted to watch TV after work. The following comment from all group homes captures clients’ embedded attitudes towards PA/inactivity: *“*Most of them just like sitting and watching TV*”.* However, “Some are willing to do physical exercise, [but] one-third won’t, even when forced” (GHA). Other groups variously reported about client disinterest in PA and their habitual physical inactivity; for example, (a) “Those who don’t like to walk won’t go out no matter what. They would [rather] spend time watching TV in their rooms or playing card games”. (GHC with outdoor facilities); (b) “They love to watch TV. It’s not about games [offered] being uninteresting; it’s just that they choose to watch TV. It’s just their ingrained habit and it takes time to change” (GHD); (c) “It’s hard to ask those who are physically inactive to join a sport program; they will only do indoor physical activities with air-conditioning. They are not interested in outdoor ones” (GHD); and (d) “Ten residents joined gateball training for 8 weeks. Afterwards, most of them said it was hard. Therefore, the program was discontinued” (GHD).

Another issue related to the characteristic of the residents was a concern of their advancing age, a major threat towards PA promotion as shared by those three group homes that had been longer in operation. Aging residents have multi-morbidities such as reduced mobility or increased joint pain, and these health conditions would possibly have led to further immobility and could further impede the rehabilitative progress from diseases.

#### 3.2.2. Resident Cognition

In a follow-up to the PA intervention, staff were asked about such disinterest and if people with ID and their cognizance about the personal benefits of a physical active lifestyle and if they could apply it their daily life. One staff member (GHC) said that “they could not [apply, do it]. We asked them the benefits of doing physical exercises, no one replied”. Similarly, when asked about using peer support in prompting PA, staff (GHC) commented that people with ID could not comprehend the meaning of peer encouragement in PA participation, and “they might perceive verbal persuasion among peers as a type of reprimand”.

#### 3.2.3. Staff Prior Ineffectiveness 

Failed experiences of staff in promoting PA could be an impediment to future health promoting action. For example, two staff at GHB trialed a PA program for six obese residents. (Exercises were trialed for 2 months, twice per week, 45 min in duration.) Feedback from activity leaders was that it was extremely difficult to operate; the program was subsequently terminated. Involved staff were disheartened with the failed experience, as inferred in the statement:

*Previously we had a yearly plan to set for residents to do physical exercise after work, say for around 30 min. But the outcome was that we didn’t see anyone showing up [for the activity].*

(GHB)

#### 3.2.4. Physical Context

The residential setting itself was often perceived as a drawback in PA promotion. Although lack of physical space of OH settings (only a single several-story building) could be solved by taking residents outside; positive intentions by staff were hampered by restricted staff numbers with implications for resident safety. At GHA, there was “limited space for PA. Although there is a basketball court outside the dormitory, we don’t have the manpower [sic] to take them outside. We worry that they may run away. Therefore, we cannot take too many people there”. Similarly, staff at GHC reported “not daring to organize hiking; safety is the first priority”. 

### 3.3. Opportunities

Opportunities are external factors having positive impact onto PA promotion, which included parental support, external funding, and external support. Group homes sought parental support by organizing parental educational talks. As commented by staff (GHB), group home had increased the priority of PA promotion because of parental advocacy, which emerged from their program review. Although generally anxious about safety, parents at GHD also agreed to their resident child joining PA programs.

#### 3.3.1. Funding

External funding came from two sources: government subsidy and charity funds. Since 2015, residential homes have been given an additional government subsidy to hire program/activity workers for rehabilitative purposes in order to combat health issues associated with the aging residential population [36]. Hence, program-related staff were motivated and had incentive to promote additional PA programs. In addition, funding was spent (GHB) or would be spent in purchasing newer fitness equipment (GHD). Furthermore, group homes were eligible to apply for external charity funds to organize different physical activities. As the newest home, admitting more younger-age residents, GHD had gained funds to organize short-term exercise programs such as hiking trips, Thai boxing, and lion dance classes. Educational sessions were conducted for parents so that they could encourage their residents to lead healthy lifestyles (GHA). Moreover, external agencies have also been engaged to deliver staff development sessions and practicum, in areas of concern such as fall prevention and fall prevention exercise. In one case, selected personnel gained additional professional development with the view that they could take up instructional roles in future PA programs (GHD). Staff considered that such competency training was a professional asset in promoting PA, as evidenced by, “We have some staff getting seated Tai-chi training and … now [lead] instruction … [in a] seated Tai-chi program for our residents” (GHA).

#### 3.3.2. University Mentorship

All four homes were linked to the sport-related department of the host university through a student internship program whereby exercise-leading trainees offered programs to ID residents and available staff. As indicated by GHB, they planned to apply for funding to host university to conduct staff training with these stated goals, “I think my staff would like to know how to set a program, how to play or have the experience of playing”.

### 3.4. Threats 

In contrast to the identification weaknesses that could be resolved within the institution, threats were those elements impacting on PA promotion, which might be viewed as external to the home. 

#### 3.4.1. Staff Availability and Qualifications

This included availability of qualified staff, aging residents with older-age parents, parental-home environment, and surrounding physical environment of the group home. Specific only to GHA was the manager mentioning difficulty in hiring staff for care-taking roles because caring for aging residents involved physically demanding work. Along with their residents, the current caretakers are also aging. As staff members retired, management had difficulty in getting personnel to take up these duties. Indeed, with the aging population of HK, hiring appropriate staff could become a wider issue.

#### 3.4.2. Advancing Age of Residents

For GHA, GHB, and GHC, having been longer in operation and having more older-age residents means, “their parents are in more advanced-age, hence parents don’t join any leisure activities” (GHC). On the other hand, when residents go back their parental home on weekends, “their parents will give them a lot of foods, perceived that residents received inadequate foods at group home” (GHD). Hence, staff commented that individualized exercise program offered to the obese residents might not be successful in weight reduction because of the influence of parental feeding practices. 

Obesity is prevalent among aging residents with ID, and individualized or group PA programs were offered to the residents. Group home staff members considered that aging residents tend to spend time “sitting” because of their declining health conditions and aging-related diseases (e.g., obesity, dementia, cancer).

#### 3.4.3. Location of Residence

GHB was located in a highly-densely populated urban area and had no public recreation park or activity center within 250 m radius in proximity. Generally, open space for hiking was over 3 km away. This was a hindrance for this group home in using neighborhood physical activity facilities. Another major hindrance for GHB was that their physical activity room had been converted to additional dormitory accommodation to cater for new admissions.

## 4. Discussion

From the analysis of staff perceptions of PA among their clients, it was evident that staffers were aware of positive (strengths and opportunities) and negative (weaknesses and threats) influences on PA engagement. These influences are associated with characteristics of residents, staff, and residential home.

### 4.1. Characteristics of Residents

Motivation, obesity, age, and interests were interactive influences on PA involvement of these HK residents. Consistent with previous research, resident low motivation and low self-efficacy towards PA, preference of sedentary lifestyle studies, and feeling of exhaustion after PA [21,37,38,39] were found to be weaknesses and barriers to PA participation. Extensive support is required to care for aging residents [40]; thus, staff shortages drained resources from PA promotion. Client age is a threat to PA participation. Aging and disability are challenges consistent with existing literature [38,40] and draw heavily on human resources at the institutionalized level. In contrast, the group home housing younger-age residents found opportunities to offer diversified sport programs catering to their interest. Peer influence on younger-age residents and staff support (strength) was seen as important strategy in PA promotion [41]. It would seem critical to increase younger-aged individuals’ PA levels so that they might attain better health before advancing age. Staff saw professional development in this area as necessary. See Table 3 for potential strategy to eliminate a “weakness” by creating an “opportunity” to build program quality.

### 4.2. Characteristics of Staff

Unsuccessful prior experience of staff in conducting PA programs was implicated as a significant barrier to PA promotion where a small group exercise training program could not be sustained because of reported participants’ low motivation and other perceived obstacles. Professional knowledge of, and personal competence, in activity skills can be either a strength or a weakness in staff promotion of PA. Thus, some staffers when frustrated gave up and no longer offered PA opportunities. As congruent with a previous study [16] others, perceiving themselves as not competent to ensure residents’ safety in PA of potential high risk. Acknowledging this weakness, staff saw additional training as necessary. After professional development, staff confidence became an internal asset (as a competent activity instructor) and a strength when including appropriate activity (seated Tai-chi) in the residents’ routine. Other staff-related factors such as persistence and interpersonal engagement with their clients doing activity together was shown [22], because role models [42,43] can become strength factors in supporting PA participation. In particular, increasing resident motivation through positive verbal encouragement and visible rewards [16,20] can facilitate PA engagement among residents.

During the PA program implementation at intervention sites (GHA and GHB), staff of these homes had first-hand observation of how to lead a group exercise class incorporating music, toys, and slow/fast tempo of movements. These strategies heightened participants’ interest. This experience could have strengthened professional knowledge in PA program content selection. However, the withdrawal of the intervention program has the potential to degrade this strength to a weakness through possible re-emergence of staff feelings of incompetence to take up the instructional role of conducting group exercise classes. Thus, the intervention program may be unsustainable upon the withdrawal of the intervention program team. (See Table 3 for a potential strategy to generate an opportunity.)

### 4.3. Characteristics of Residential Homes

As with western-based literature [10,11], our results demonstrate residential home context is particularly influential in the quality of PA programs. An ultra-metropolitan city, HK has limited indoor activity space (especially OHs). Local context was viewed as a weakness, as it restricted the offering of group exercise programs. However, when fitness equipment was available, individualized rehabilitative exercise programs were facilitated. To compensate for lack of facilities, additional resources and support are needed. Indeed, a lack of available resources impedes participation [22,43,44,45]. Since 2015, group homes for the moderately mentally handicapped in HK have been given additional financial funding by the government to increase residential care support for their aging residents (i.e., to hire program/activity workers for rehabilitative purposes) [46]. Soliciting external funding is an opportunity and an enabler to promote sport programs as evidenced by GHD’s application to a charities fund to finance culturally relevant PA programs and, in addition, seeking external support from a local university for a PA promotion program. On the other hand, institutional policy influences PA promotion. A successful incidence on increasing attendance of exercise classes was cited. It involved a policy change from voluntary to mandatory participation and the timetable switch from exercise time from pre-dinner to after-breakfast. These are effective strategies in eliminating a “weakness” of an ineffective program (voluntary participation and undesirable program timing) so that a “strength” can be cultivated to sustain a program. (See Table 3 for illustration.)

### 4.4. Recommendations for Practice

In terms of professional practice, these HK findings indicate specific health behavioral techniques that can be adopted to break the residents’ habit of sedentariness, low self-efficacy, and low motivation and to eliminate weaknesses to PA engagement, which are generally similar to Western findings. As evidenced from our study, we recommend the following strategies to consolidate strengths and capitalize opportunities for quality PA program outcomes. Firstly, use the reward of social recognition to increase the residents’ extrinsic motive; for example, set-up an honors board depicting photos of the most improved residents based on their weekly behavioral assessment or visual reward such as a badge. Secondly, use music and rhythmic movements in heightening participants’ interest in doing PA. Exercises comprising physical movements can be adapted from diversionary therapy [47], which has worked well on abled aging individuals and people with disabilities using chair calisthenics with props (e.g., colorful scarves, gloves, balls) and music [48]. Thirdly, set-up institutional policy for embedded daily PA into group home routines. Examples are daily physical chores of light intensity by assigning duties requiring walking and mandatory morning exercise session with music. Fourthly, institutional policy of sheltered workshop incorporating short exercise breaks (with varieties of simple repetitive creative movements such as stand-up and sit-down and movement sequences involving hands and body) into residential daily work schedule may be instrumental in interrupting seated work routines. Fifthly, we suggest that the institutions collaborate with Special Olympics HK on PA promotion. Lastly, seek external support to improve PA program, such as having university faculty design and produce supplementary PA resources. A strategy to improve staff quality is for the university sector to offer professional training programs in adapted PA for special populations at the certificate, degree, and/or post-graduate level.

## 5. Conclusions

A SWOT analysis provided important insights into influences on the quality of PA programs for residents with ID, included characteristics of resident, staff, residential institution, and external resources. Their increasing age and low motivation are impediments to PA engagement of adults with ID. Staff competence and prior unsuccessful experience in promoting PA are also implicated. The PA program quality is mediated by staff quality of personal interaction with their clients, their commitment in encouraging adults with ID to join, and persistence in exercise engagement as well as staff seeking external resources and support. These results have elucidated best practice in PA promotion among Chinese residents with ID in the east Asian context of HK. In order to promote active lifestyle of such residents, removing or minimizing weaknesses and threats concerning personal and environmental barriers and capitalizing strengths and opportunities for enabling environments are essential in the provision of quality PA programs. Future PA intervention programs should be conducted to evaluate the effectiveness of exercises, incorporated with strategies of enhanced motivation by rewarding participation, provision of active staff support, and varied game-like creative movement.

One methodological strength of this study was the use of a SWOT analysis to determine salient factors influencing the provision of PA programs for adults with ID. Although different studies have identified barriers and facilitators affecting low level of PA in people with ID [14], to our knowledge, there were only few studies analyzing the influencing factors impacting directly on the quality of PA programs. Another strength was the use of a CF to review data coding and discuss the quality of data analysis in a situation of potential bias wherein researchers were also involved in planning, implementing, and evaluating the PA promotion program. However, potential limitations of the study were identified. Because two group homes were involved in the intervention study, their staff might be inclined to give favorable feedback on the intervention program. In addition, the selected group homes mainly admitted residents with mild and moderate ID; hence, results may not be applicable to group homes that admit individuals with severe ID.

## Figures and Tables

**Table 1:** Characteristics of four group homes for people with mild and moderate intellectual disabilities (ID).

Group Home ^a^ (Inception Year)	Admitted Residents	Location	Total Indoor Physical Activity Areas (m^2^)	PA Venues (Outdoor)	Location of Sheltered Workshop	Remark
OH ^b^^,c^ – GHA (1995)	55 mild and moderate ID	Indoor only, 1 floor (inside a public housing estate block)	135	- 2 Multi-purpose room	Same building	Public outdoor sport facilities within 50 m
OH ^b,c^ – GHB (2001)	55 mild and moderate ID	Indoor only, 3 floors within a private and commercial building ^d^	295	- 1 Multi-purpose room - Fitness room	Same building	Public sport facilities outside 250 m radius (road crossings)
NH ^b^ – GHC (2010)	106 (34 moderate, 72 severe ID) ^e^	Standalone building and outdoor area	974	- Multi-purpose hall (Outdoor: adventure corner, climbing wall)	Same building	Enclosed compound with outdoor space
NH ^b^ – GHD (2014)	69 mild and moderate ID ^f^	Standalone building and outdoor area	438 (indoor) 1654 (outdoor track)	- Activity room - Fitness room (Outdoor: badminton, basketball court, climbing facility, jogging, proprioceptive corner, soccer-hard court)	Requires bus transport	Enclosed compound with outdoor space

^a^ GHA and GHB and GHC and GHD were managed by two different non-government agencies separately; ^b^ OH – old-smaller home; NH – new-larger home; ^c^ About 2/3 residents of GHA and GHB had health or aging-related complications such as hypertension, diabetes, high cholesterol; ^d^ Residents used elevators and stairways to get access to different floors; ^e^ GHC had an additional 94 residents of physical disabilities; ^f^ GHD, newly operated, had mostly younger-age adults with ID (average of 20–30 years old, admitting school leavers of 16–17 years old from a nearby special school for mild and moderate ID).

**Table 2:** List of physical activities offered by group homes.

	GHA	GHB	GHC	GHD
Regular Physical Activities				
Basketball			X ^c^	
Dance class (video)				X ^c^
Exercises (badminton, stationary cycling)			X ^c^	X ^a^
Individualized fitness exercise (targeted residents) ^e^	X ^b^	X ^b^	X ^b^	
Hiking trip (weekends)	X ^d^			X ^d,k^
Morning exercise (20 min, video)		X ^a^		
Seated Tai-chi (weekends)	X ^c^			
Small group fitness exercise (targeted residents) ^f^				X ^c^
Swimming classes (weekends)				X ^d^
Walking trip to community (weekends)	X ^c^			X ^c^
Walking trip to supermarket	X ^c^	X ^c^		X ^c^
One-off Physical Activity Program (mostly 1X per week for 8 weeks, led by group home staff ^g^, part-time instructors ^h^, or external organizations ^i^)				
Fall prevention training		X ^i^		
Gate ball			X ^h^	
Jogging training		X ^i^		X ^i^
Lion dance			X ^h^	X ^h,k^
Multi-ball games (offered twice) (basketball, handball, soccer)			X ^g^	
Small group exercises/games	X ^i^	X ^i^	X ^i^	
Taekwondo				X ^h^
Thai boxing				X ^h,k^
Video games of bodily movements			X ^g^	

^a^ Daily; ^b^ 2X per week; ^c^ 1X per week or 1X per two weeks; ^d^ seasonal, 1X per few months; ^e^ for overweight/obese or inactive ones, designed by physiotherapist/occupational therapist, 10 to 20 min duration on treadmill, stationary cycling, or calisthenics exercises; ^f^ for 10 highest BMI; 1-h outdoor games or indoor fitness activities; ^g^ led by group home staff; ^h^ led by part-time instructor; ^i^ led by external organization; ^j^ led by group home’s staff; ^k^ applied external fund; ^l^ future plan.

**Table 3:** Examples of potential strategies for physical activity (PA) promotion to generate strengths and opportunities based on a SWOT analysis.

	Positive Influence onto PA Promotion	Negative Influence onto PA Promotion
Internal Factors (within the scope or control of the residential policy/program)	Strengths	Weaknesses
	Staff recognize need for ideas and skills in leading PA	(1) Resident characteristic: ingrained sedentary habit
		Potential Strategy
		Offer staff training to lead PA programs
	Institutional policy makes PA participation unavoidable	(2) Home characteristic: voluntary participation with program scheduled at a desirable time
		Potential Strategy
		Change institutional policy: mandate PA participation; schedule PA at a time when clients are not tired from work; lock dormitory doors when PA programs are scheduled
External Factors (outside the direct control of the residential policy/program)	Opportunities	Threats Availability of quality staff
	Supply a larger pool of trained healthcare workers	Potential Strategy
		Seek external support with agencies to offer certificate/degree courses in adapted physical activities or healthcare for special populations, which are recognized by the social services sector.
